# Decentralized collaborative multi-institutional PET attenuation and scatter correction using federated deep learning

**DOI:** 10.1007/s00259-022-06053-8

**Published:** 2022-12-12

**Authors:** Isaac Shiri, Alireza Vafaei Sadr, Azadeh Akhavan, Yazdan Salimi, Amirhossein Sanaat, Mehdi Amini, Behrooz Razeghi, Abdollah Saberi, Hossein Arabi, Sohrab Ferdowsi, Slava Voloshynovskiy, Deniz Gündüz, Arman Rahmim, Habib Zaidi

**Affiliations:** 1 Division of Nuclear Medicine and Molecular Imaging, Geneva University Hospital, CH-1211 Geneva 4, Switzerland; 2 Department of Theoretical Physics and Center for Astroparticle Physics, University of Geneva, Geneva, Switzerland; 3 Institute of Pathology, RWTH Aachen University Hospital, Aachen, Germany; 4 Department of Computer Science, University of Geneva, Geneva, Switzerland; 5 HES-SO, University of Geneva, Geneva, Switzerland; 6 Department of Electrical and Electronic Engineering, Imperial College London, London, UK; 7 Departments of Radiology and Physics, University of British Columbia, Vancouver, Canada; 8 Department of Integrative Oncology, BC Cancer Research Institute, Vancouver, BC Canada; 9 Geneva University Neurocenter, Geneva University, Geneva, Switzerland; 10 Department of Nuclear Medicine and Molecular Imaging, University of Groningen, University Medical Center Groningen, Groningen, Netherlands; 11 Department of Nuclear Medicine, University of Southern Denmark, Odense, Denmark

**Keywords:** PET, Attenuation correction, Deep learning, Federated learning, Distributed learning

## Abstract

**Purpose:**

Attenuation correction and scatter compensation (AC/SC) are two main steps toward quantitative PET imaging, which remain challenging in PET-only and PET/MRI systems. These can be effectively tackled via deep learning (DL) methods. However, trustworthy, and generalizable DL models commonly require well-curated, heterogeneous, and large datasets from multiple clinical centers. At the same time, owing to legal/ethical issues and privacy concerns, forming a large collective, centralized dataset poses significant challenges. In this work, we aimed to develop a DL-based model in a multicenter setting without direct sharing of data using federated learning (FL) for AC/SC of PET images.

**Methods:**

Non-attenuation/scatter corrected and CT-based attenuation/scatter corrected (CT-ASC) ^18^F-FDG PET images of 300 patients were enrolled in this study. The dataset consisted of 6 different centers, each with 50 patients, with scanner, image acquisition, and reconstruction protocols varying across the centers. CT-based ASC PET images served as the standard reference. All images were reviewed to include high-quality and artifact-free PET images. Both corrected and uncorrected PET images were converted to standardized uptake values (SUVs). We used a modified nested U-Net utilizing residual U-block in a U-shape architecture. We evaluated two FL models, namely sequential (FL-SQ) and parallel (FL-PL) and compared their performance with the baseline centralized (CZ) learning model wherein the data were pooled to one server, as well as center-based (CB) models where for each center the model was built and evaluated separately. Data from each center were divided to contribute to training (30 patients), validation (10 patients), and test sets (10 patients). Final evaluations and reports were performed on 60 patients (10 patients from each center).

**Results:**

In terms of percent SUV absolute relative error (ARE%), both FL-SQ (CI:12.21–14.81%) and FL-PL (CI:11.82–13.84%) models demonstrated excellent agreement with the centralized framework (CI:10.32–12.00%), while FL-based algorithms improved model performance by over 11% compared to CB training strategy (CI: 22.34–26.10%). Furthermore, the Mann–Whitney test between different strategies revealed no significant differences between CZ and FL-based algorithms (*p*-value > 0.05) in center-categorized mode. At the same time, a significant difference was observed between the different training approaches on the overall dataset (*p*-value < 0.05). In addition, voxel-wise comparison, with respect to reference CT-ASC, exhibited similar performance for images predicted by CZ (*R*^2^ = 0.94), FL-SQ (*R*^2^ = 0.93), and FL-PL (*R*^2^ = 0.92), while CB model achieved a far lower coefficient of determination (*R*^2^ = 0.74). Despite the strong correlations between CZ and FL-based methods compared to reference CT-ASC, a slight underestimation of predicted voxel values was observed.

**Conclusion:**

Deep learning-based models provide promising results toward quantitative PET image reconstruction. Specifically, we developed two FL models and compared their performance with center-based and centralized models. The proposed FL-based models achieved higher performance compared to center-based models, comparable with centralized models. Our work provided strong empirical evidence that the FL framework can fully benefit from the generalizability and robustness of DL models used for AC/SC in PET, while obviating the need for the direct sharing of datasets between clinical imaging centers.

**Supplementary Information:**

The online version contains supplementary material available at 10.1007/s00259-022-06053-8.

## Introduction

PET is widely used for in vivo quantification of physiological processes at the molecular level [1]. The introduction of hybrid imaging, in the form of PET/CT has thrived its adoption in a clinical setting, particularly for oncological applications [1]. Corrections for physical degrading factors mainly linked to the interaction of annihilation photons with matter, such as attenuation and Compton scattering, are needed to achieve the full potential of quantitative PET imaging [2]. During the image formation process, a significant number of annihilation photons undergo photoelectric absorption and multiple Compton interactions with underlying material along their trajectory (patient body, scanner hardware, etc.) before reaching the PET detectors [2, 3]. Attenuation and scattering interactions result in undetected annihilation events and the recording of anomalous coincidences, respectively [4]. This leads to a large tracer uptake quantification bias. It has been reported that a fraction of around 30–35% of all detected events in 3D brain scanning are recorded from scattered photons, while this fraction exceeds 50–60% in whole-body scanning [5]. The probability of photon interactions increases either with the traveling distance (patient’s size) or the electron density of the medium [4]. Hence, for an effective attenuation/scatter correction (AC/SC) of PET images, a prior knowledge of the attenuation map at 511 keV through the traveling medium is required [4].

The problem of AC/SC to achieve quantitative PET imaging has been relatively successfully resolved following the commercial emergence of hybrid PET/CT modality where CT-based correction algorithms are commonly implemented on commercial systems [4, 6]. However, AC and SC remain challenging on PET/MRI and PET-only scanners [7, 8]. Unlike PET/CT, direct attenuation correction in PET/MRI is not straightforward owing to the lack of direct correlation between MR signals, i.e., proton density and time-relaxation properties of tissues and electron density [8]. Hence, various strategies have been devised for MRI-guided AC/SC, including bulk segmentation, atlas-based algorithms, and emission-based techniques. Although these methods improve the quantification accuracy of PET images, they are affected by the misclassification of tissues (segmentation-based approach), as well as inter/intra-subject variability of MR images for co-registration to the best-fitted atlas model (atlas-based approach) [8]. Furthermore, in PET-only scanners, emission-based algorithms that estimate directly the attenuation map from the emission data, time-of-flight (TOF) information, and anatomical prior knowledge have been proposed [9, 10].

The past decade has witnessed significant progress in the development and implementation of artificial intelligence (AI)-based methods in different areas of medical image analysis, e.g., detection, segmentation, classification, regression, and outcome prediction [11–14]. Several AI-based algorithms, in particular deep convolutional neural networks, have been developed to address the limitations of conventional attenuation correction techniques, demonstrating significant benefits in terms of improved image quality and quantitative accuracy of PET imaging [15, 16]. In this context, four main learning-based approaches for AC/SC of PET data, including (i) the generation of synthesized CT from MR images [17], (ii) generating synthesized CT from non-corrected PET images [18], (iii) predicting the scattered component from emission information (TOF, event position) in either the image or sinogram domain [10, 19], and (iv) generating directly AC/SC PET images from non-attenuation/scatter corrected images [20]. Although, a number of studies reported promising performance of deep learning (DL)-based algorithms within an acceptable clinical tolerance, the size of training and testing datasets is a major limitation of these methods [21]. To build a generalizable and trustworthy DL model, a large multicenter dataset is required to tune millions of model parameters [22–24]. However, the sensitivity of medical images, and the ensuing ethical/legal considerations and regulations, challenge gathering large datasets to feed such data-hungry algorithms [22–24]. To address this issue, federated learning (FL), initially developed for mobile technologies, is being increasingly considered in the healthcare domain [4].

A single hospital, often, cannot provide a sufficient number of samples, as required for successful training of machine learning models with acceptable accuracy, generalizability, and trustworthiness [22–25]. As such, it may not be feasible to train a high-quality model for PET AC/SC images based on a limited sample dataset available from a single hospital. Moreover, all hospitals do not have infrastructures and expertise for machine learning model developments. One strategy involves collecting data from different hospitals to train a more accurate model. However, this approach is challenged by various privacy regulations and policies on data sharing. FL techniques enable the collaborative training of machine learning models among multiple parties without exchanging the local data to preserve privacy and solve the concerns of data users and data owners [22–24].

A typical FL protocol consists of three main components: (i) the manager (e.g., trusted server), (ii) participating parties as data owners (e.g., hospitals and departments), and (iii) computation-communication framework to train the local and global models [26]. Depending on the parties, FL protocols can be divided into two settings: (i) cross-device FL, where the parties are edge devices; and (ii) cross-silo FL, where the parties are reliable organizations (e.g., hospitals). In designing an FL system, one needs to consider three properties regarding the participating parties namely, (a) computational and storage capacity of the parties, (b) stability and scale of the parties, and (c) data distribution among the parties [27, 28]. The manager (trusted server or party) supervises the training procedure of the global model and manages the communication between the data owners and itself. To produce an accurate model, the stability and reliability of the server need to be guaranteed [27, 28]. In the cross-device setting, various solutions have been proposed to increase the reliability of the system [29–32]. Fortunately, in the cross-silo setting, organizations have powerful computational machines, better facilitating FL [29–32]. Hence, one possible option is to consider one of the organizations as the manager of the FL model [27, 28]. Alternatively, the organizations can act in a fully decentralized setting. In this setting, all the participated parties communicate with each other directly [29–32].

Collaborative models could be trained in a decentralized manner using an FL framework without exchanging data between the different centers/hospitals [27, 28]. In recent years, FL-based DL models have been applied to multi-institutional data for different medical imaging tasks, including image segmentation [33–35] and abnormality detection and classification [36–38]. The main contribution of the present study is to propose, implement, and assess a robust FL algorithm for attenuation/scatter correction of PET data to achieve a generalized model using a limited data obtained from each center without direct sharing of data amongst the different centers. The hope is to propose this development for potential applications on standalone CT-less PET scanners or enhanced quality assurance in PET/CT scanners.

## Materials and methods

### PET/CT datasets

Non-attenuation-corrected and CT-based attenuation-corrected ^18^F-FDG PET images of 300 patients were included in this study. The dataset were acquired at 6 different centers, each providing 50 patients acquired on various PET scanners, using different image acquisition and reconstruction protocols across the different centers, more information of dataset is provided in Table 1 [20, 39–47]. All images were reviewed to include only high-quality and artifact-free PET images. PET images were converted to standardized uptake values (SUVs) for both corrected and non-corrected images.

**Table 1:** Patients demographics and PET/CT image acquisition and reconstruction settings across the six different centers

		Centre 1	Centre 2	Centre 3	Centre 4	Centre 5	Centre 6
Demographic	Sex (F/M)	15/35	17/33	19/31	22/28	6/44	21/29
	Age	54 ± 22.7	62.6 ± 8.8	63.9 ± 12.2	68 ± 9.4	58.2 ± 9	52.6 ± 20.2
	Weight	69.1 ± 15.9	68.2 ± 18.4	77.3 ± 18.7	74.5 ± 16.1	84.3 ± 18	70.2 ± 23.3
Scanners	Manufacture	GE	GE	GE	GE	GE	Siemens
	Model	Duo	LS	ST	Discovery 690	RX	Biograph
CT acquisition	Average tube current	115.7 ± 9.2	120.6 ± 41	149.2 ± 51.9	98.3 ± 61	264.1 ± 41.9	176 ± 32.0
	kVp	130 ± 0	135 ± 8.8	134 ± 9.3	134 ± 9.3	119.2 ± 4	130± 0
PET acquisition and reconstruction parameters	Injected dose	487.2 ± 72.9	514.3 ± 118.1	549.7 ± 95.2	425.5 ± 91.2	448.9 ± 121.8	373.9 ± 92.6
	Time to scan	75.7 ± 18.9	72.1 ± 25.5	75.2 ± 17.6	73.1 ± 18.8	86.7 ± 13.6	97.6 ± 13.9
	Time Per Bed	2.6 ± 0.5	4.6 ± 1	3.6 ± 0.6	2.4 ± 1	3.1 ± 0.3	3.1 ± 0.4
	Scatter Correction	Model-based	Convolution subtraction	Convolution subtraction	Model-based	Model-based	Model-based
	Reconstruction	OSEM	OSEM	OSEM	VPHD, VPHDS	OSEM	OSEM + PSF
	Matrix size	256 × 256	128 × 128	128 × 128	192 × 192	128 × 128	168 × 168
	Slice thickness	3.4	4.3	3.3	3.5	3.3	3
	Slice numbers	14,598	10,647	13,683	16,282	11,002	26,210

### Image preprocessing

We converted all PET images to SUV values and resampled both NAC and CT-ASC PET images to the same voxel spacing (3 × 3 × 4 mm^3^) and finally normalized by empirical values of 3 and 9 for NAC and CT-ASC, respectively. To harmonize the intensity range of PET images across the different centers, the voxel values of both non-AC and CT-AC PET images were converted to SUV. Subsequently, non-ASC and CT-ASC PET images were normalized by SUV factors of 3 and 9, respectively. In this way, the intensity range of all PET images across the different centers was between 0 and 5.

### Global FL training

In typical machine learning problems, the goal is to minimize an appropriate loss function ${F}\left(\uptheta \right)$, where $\uptheta \in {\mathbb{R}}^{d}$ denotes the parameters of the model. The loss function ${F}\left(\uptheta \right)$ represents the average of empirical loss functions over the available data samples with respect to model parameter $\uptheta$. A common approach to minimize the loss function ${F}\left(\uptheta \right)$ is to use the iterative Stochastic Gradient Descent (SGD) algorithm. The idea of federated DL originates from the fact that SGD allows parallelization [26, 48–53]. Hence, one can optimize a machine learning model using *distributed* SGD. The framework is as follows.

Consider the FL system with $K$ parties, where the $k$-th party has a local training dataset ${{\mathcal{D}}_{k} = \left\{{X}_{i} , {Y}_{i}\right\}}_{i=1}^{{N}_{k}}$, where ${X}_{i}$ and ${Y}_{i}$ are the feature vector and the ground-truth label vector, respectively, and ${N}_{k}$ is the sample size available at party$k\in \left\{\mathrm{1,2}, ..., K\right\}$. Let all parties have $N=\sum_{k=1}^{K}{N}_{k}$ samples, and let ${F}_{k}\left(\uptheta \right)$ denotes the local objective function of the $k$-th client, i.e., we have:

1 $${F}_{k}\left(\uptheta \right) = \frac{1}{{N}_{k}} \sum_{i=1}^{{N}_{k}}\mathcal{L}\left(\theta ;\left({X}_{i}, {Y}_{i}\right)\right) ,$$

where $\uptheta \in {\mathbb{R}}^{d}$ denotes the model parameters to be optimized and $\mathcal{L}(.;.)$ is the specific loss function. As an example, one can consider the mean square error loss function${\mathcal{L}\left(\Theta ;\left({X}_{i}, {Y}_{i}\right)\right)=\frac{1}{2}\Vert {y}_{i}- \widehat{{y}_{i}}\Vert }_{2}^{2}$, where $\widehat{{y}_{i}}$ is the corresponding predicted label, and $\Vert .\Vert$ is the ${l}_{2}$-norm. In this case, we consider the *global* optimization problem of our FL system as follows:

2 $$\underset{\uptheta }{\mathrm{min}}\left(F\left(\theta \right)=\sum_{k=1}^{K}\frac{{N}_{k}}{N}{F}_{k}\left(\theta \right) \right) ,$$

In this framework, the local objective function for each center is weighted by the fraction of data emerging from that center. In order to solve the above optimization problem, the SGD algorithm can be utilized. Therefore, at the $t$ th iteration, each party computes local gradients using the SGD method, and sends them back to the manager (server) for aggregation and updating. Let $\nabla {F}_{k}\left({\theta }_{t}\right)$ denote the local gradient on the local data of the $k$ th party at the $t$ th iteration. Let $\eta$ represent the learning rate and let ${\theta }_{t}$ denote the model at *t*th iteration. The server aggregates and updates the model parameters as follows:

3 $${\theta }_{t+1}\leftarrow {\theta }_{t}- \eta \sum\nolimits_{k=1}^{K}\frac{{N}_{k}}{N}\nabla {F}_{k}\left({\theta }_{t}\right) ,$$

Note that in the case of massive datasets, the SGD becomes prohibitively demanding. Hence, the parameter vector is updated with the stochastic gradient:

4 $${\theta }_{t+1}= {\theta }_{t}- {\eta }_{t}G\left({\theta }_{t}\right)$$

where $${\mathbb{E}}\left[G\left({\theta }_{t}\right)\right]= \nabla F\left({\theta }_{t}\right)$$.

**Fig. 1:**
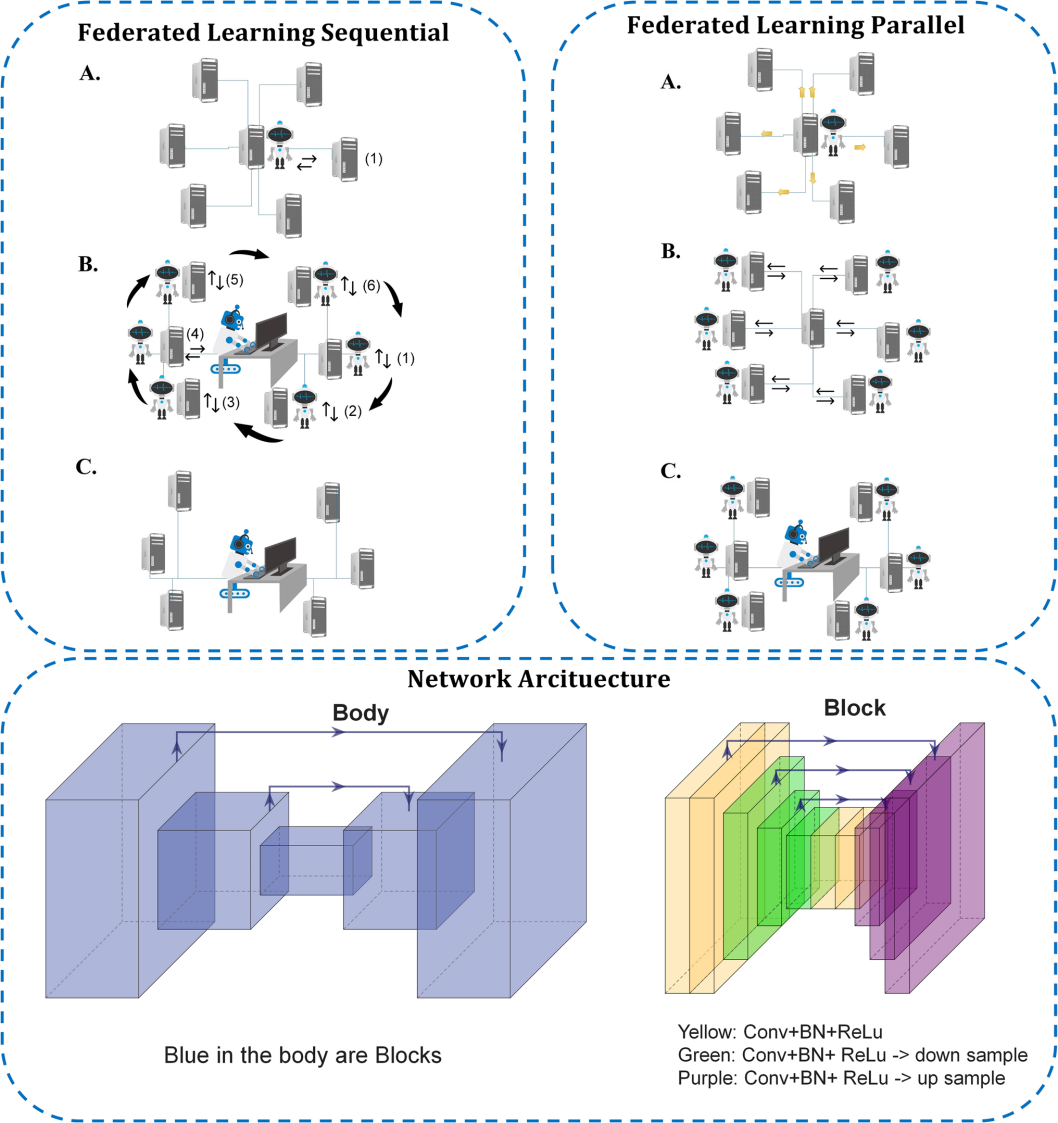
Schematic of two FL algorithms and network architectures as implemented in this study. In parallel federated learning (FL-PL), in the first step (**A**), the central global model is distributed through different centers and then (**B**) the models are trained in each center separately, and finally (**C**), local-trained models return to the central server and aggregate the results to the central global model. Steps (**A**–**C**) are repeated until the model is fully trained and converged. In sequential federated learning (FL-SQ), the model meets the data serially center-after-center. First (step **A** in Fig. 1), model training begins in one center for a predefined number of epochs, and then (step **B**) the model passes sequentially through all centers. Finally (step **C**), this process will be repeated for a predefined number of rounds to generate the ultimate model. The bottom image depicts our U2Net architecture; each blue block in the main body (left) consists of a residual U-Net (right)

We evaluate two different training strategies for our federated pipeline. In the first training approach, a server aggregates the FL workflow as summarized in Fig. 1. We refer to this first strategy as parallel federated learning (FL-PL): first (step A), the central global model is distributed through different departments and then (step B) the models are trained in each center separately, and finally (step C) the locally trained models return to the central server and aggregate the results to the central global model. Steps A–C are repeated until the model is fully trained and converges. In the second approach, referred to as sequential federated learning (FL-SQ), the model meets the data serially center-after-center. First (step A in Fig. 1), model training begins in one center for a predefined number of epochs, and then (step B) the model passes sequentially through all centers. Finally (step C), this process will be repeated for a predefined number of rounds to generate the ultimate model. FL-SQ requires longer training time since the learning procedure is sequential. As for the implementation of our experiments, all FL algorithms and DL models were implemented in TensorFlow 2.6 (details on DL models are provided below). The FL process in this work was performed on a server with multiple local GPUs similar to previous studies [36, 54–59], where each local GPU was considered as our node and center.

### Deep neural network

In this study, we used the modified U^2^-Net [60] which utilizes residual U-blocks in a U-shaped architecture. It employs a deep network supervision strategy, where the training loss includes information in all scales. Deep supervision allows to extract both local and global contextual information [60]. The advantage is that unlike the prevalently utilized U-Net based on successive down-sampling of the image and hence gradually losing high-resolution information [60], the U^2^-Net does not sacrifice the high-resolution content of the images [60], which is crucial for many image-to-image conversion tasks, such as attenuation scatter correction.

This is performed using a nested two-level U-structure inspired from the classical U-Net. The idea is to keep the general U-structure of the U-Net [61], but inside each convolutional block, it uses another structure which again has a U-shaped form with its symmetric encoder-decoder architecture [60]. This block is known as ReSidual U-block (RSU), which enables intra-stage multi-scale features to be extracted. The RSU is motivated by the classic U-Net [61] with a symmetric encoder-decoder structure. It provides a mixture of receptive fields with different sizes, which is highly desirable for fine-grained image-to-image tasks [60]. This is equivalent to drastically increasing network layers, but with the important advantage of keeping the computational and memory footprint low and hence the training procedure simple [60]. Note that the idea of having nested U-Net is different from the more common strategy of cascading multiple U-Nets together, which increases the computational burden proportional to the number of networks used [60]. The nested structure enables the U^2^-Net to extract intra-stage multi-scale, as well as aggregated inter-stage multi-level features [60]. As for the training strategy, the network further uses deep supervision, where the training loss includes information in all scales [60]. Deep supervision allows to further extract both local and global contextual information [60].

It can evoke intra-stage features in different scales depending on the depth and kernel size [60]. One can select an optional depth to achieve various single-level or multi-level nested U-shape structures [60]. Although too deep models might get too complex with respect to implementation and employment in training procedures and real-world applications. In this work, non-attenuation/scatter-corrected images were used as input to the modified U^2^-Net to generate attenuation/scatter-corrected PET images directly. The network was trained in a 2D manner with an Adam optimizer, a learning rate of 0.001, an L2-norm loss, as well as a weight decay of 0.0001. The schema of the network is depicted in Fig. 1.

### Evaluation strategy

In this study, we evaluated two federated models, referred to as FL-SQ and FL-PL, and compared their performance with the centralized (CZ) approach, wherein the data are pooled to one server. Moreover, center-based (CB) models were built and evaluated separately using only the training/test datasets from the same center. Each center’s data were divided into training (30 patients), validation (10 patients), and test sets (10 patients). A standard train/validation/test data splitting was followed for the training of all models and the results were reported on untouched test sets to avoid the risk of overfitting. There was no overlap between training, validation, and testing sets. The same patients were used for evaluation of the different non-CB models to facilitate comparison of the various models. In the three non-CB strategies, including FL-SQ, FL-PL, and CS, the models were built using a 180/60 train/validation set, and the results were reported using 60 test sets (the 10 test datasets from each of the six centers). In CB models, six different models were developed using 30/10 train/validation, and only 10 test sets from the same center were employed for model evaluation.

For model performance evaluation, voxel-wise mean error (ME), mean absolute error (MAE), relative error (RE%), absolute relative error (ARE%), and peak signal-to-noise ratio (PSNR) were computed between ground truth CT-based attenuation/scatter corrected and the predicted corrected PET images, as follows:

5 $$ME=\frac{1}{vxl}\sum\nolimits_{v=1}^{vxl}{PET}_{Predicted}(v)-{PET}_{CT-ASC}(v)$$

6 $$MAE=\frac{1}{vxl}\sum\nolimits_{v=1}^{vxl}\left|{PET}_{predicted}(v)-{PET}_{CT-ASC}(v)\right|$$

7 $$RE(\mathrm{\%})=\frac{1}{vxl}\sum\nolimits_{v=1}^{vxl}\frac{{\left({PET}_{predicted}\right)}_{v}-{\left({PET}_{CT-ASC}\right)}_{v}}{{\left({PET}_{CT-ASC}\right)}_{v}}\times 100\mathrm{\%}$$

8 $$ARE(\mathrm{\%})=\frac{1}{vxl}\sum\nolimits_{v=1}^{vxl}\left|\frac{{\left({PET}_{predicted}\right)}_{v}-{\left({PET}_{CT-ASC}\right)}_{v}}{{\left({PET}_{CT-ASC}\right)}_{v}}\right|\times 100\mathrm{\%}$$

9 $$PSNR(dB)=10{\mathrm{log}}_{10}(\frac{{Peak}^{2}}{MSE})$$

where *PET*_*predicted*_ denotes DL-based corrected PET image while *PET*_*CT-ASC*_ stands for the reference PET-CT-ASC image, and *vxl* and *v* denote the total number of voxels and voxel index, respectively. Moreover, the structural similarity index (SSIM) was calculated based on [62].

The different plots (box, bar, and scatter plots) were provided to enable different comparisons. Two-sample Wilcoxon test (Wilcoxon rank sum test or Mann–Whitney test) was used for the statistical comparison of image-derived metrics between the different training models. We corrected *p*-values using Benjamin Homberg to provide an adjusted *p*-value (*q*-value). A threshold of 0.05 was considered as the significance level of *q*-values. In addition, we used joint histogram analysis to depict the distribution of voxel-wise PET SUV correlations between the reference CT-based ASC images and different DL approaches.

## Results

**Fig. 2:**
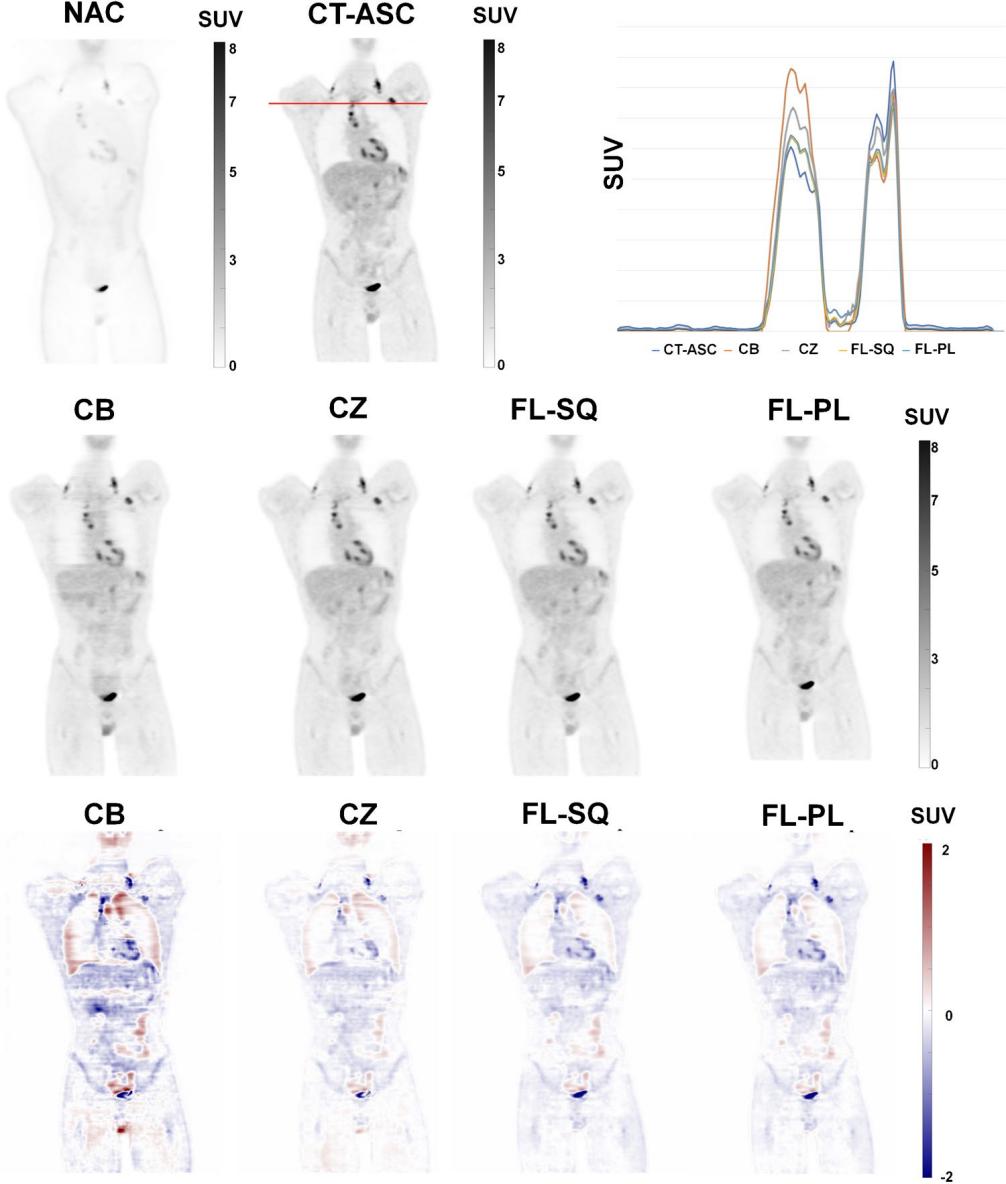
Example of non-ASC, CT-ASC, CB model, CZ model, FL-SQ model, FL-PL model, and their corresponding bias maps generated for DL models with respect to CT-based ASC (CT-ASC) images. Sequential federated learning (FL-SQ), and parallel federated learning (FL-PL), centralized (CZ), and center based (CB)

Figure 2 represents an example of non-ASC, CT-ASC, CB model, CZ model, FL-SQ model, FL-PL model, and the corresponding bias maps generated for DL models with respect to CT-based ASC (CT-ASC) images. As can be seen, the CZ-based model, FL-SQ model, and FL-PL model generated high-quality images. More examples of images for each of the centers are provided in supplemental Fig. 1.

**Fig. 3:**
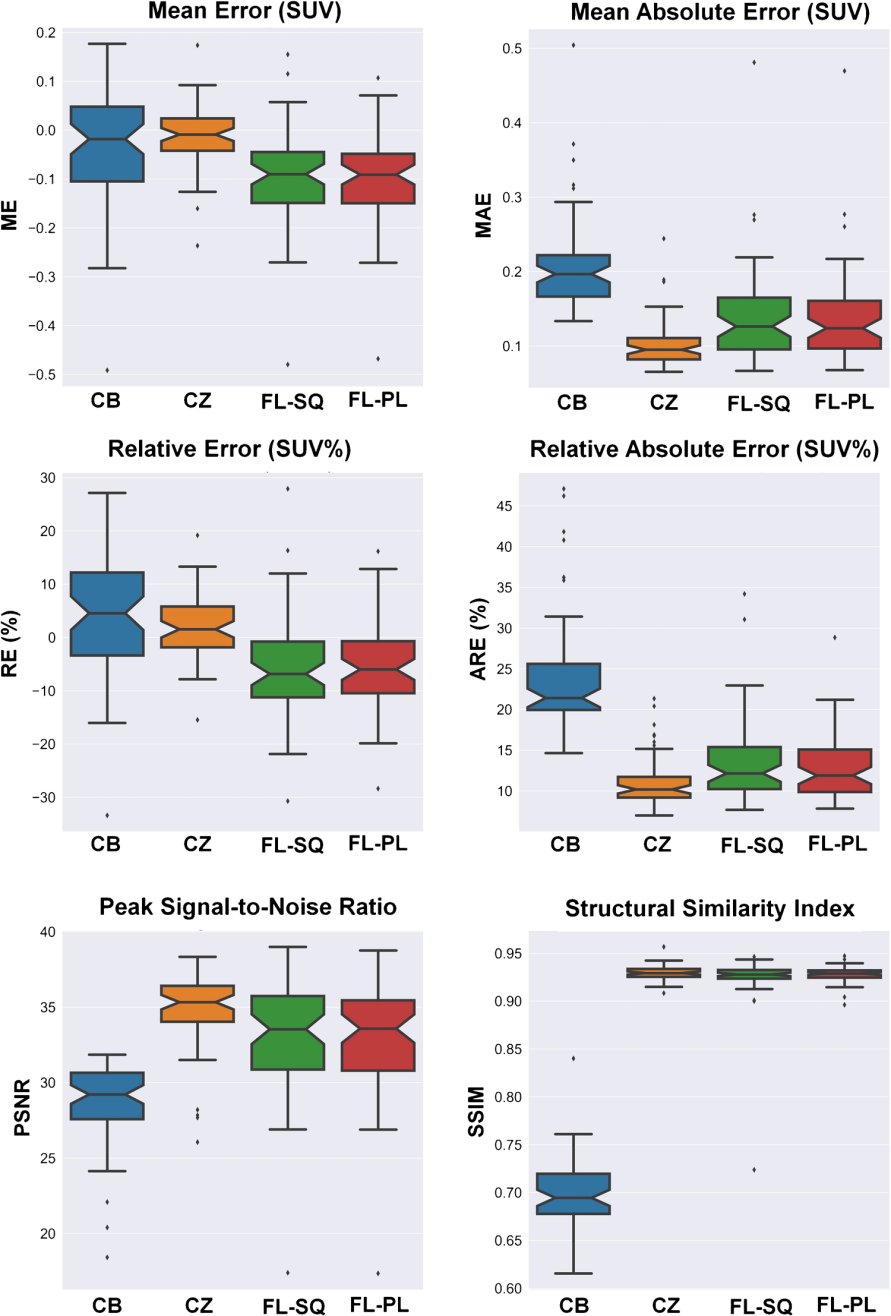
Comparison of quantitative image quality metrics, including RE (%), ARE (%), ME, MAE, SSIM, and PSNR, calculated on SUV images between different training strategies with respect to CT-ASC images serving as ground truth sequential federated learning (FL-SQ), and parallel federated learning (FL-PL), centralized (CZ), and center-based (CB)

Figure 3 compares quantitative image quality metrics, i.e., RE (%), ARE (%), ME, MAE, SSIM, and PSNR, calculated on SUV images between the different training strategies with respect to CT-ASC images serving as ground truth. As expected, the CB training strategy is the worst method in terms of quantitative analysis, resulting in the highest absolute error (MAE = 0.21 ± 0.07). The performance of the FL-based algorithms is comparable with the centralized training strategy, while the CZ method shows a lower deviation and smaller variance compared to FL-SQ and FL-PL, in terms of MAE (0.10 ± 0.03 versus 0.14 ± 0.07 and 0.14 ± 0.06, respectively). Table 2 summarizes the statistical comparisons of quantitative metrics between these four training strategies. In terms of overall structural similarity, the different approaches demonstrated comparable performance against ground truth (CZ = 0.93 ± 0.01, FL-SQ = 0.93 ± 0.01, and FL-PL = 0.92 ± 0.03), except for the CB achieving an SSIM of 0.70 ± 0.04. Table 3 summarizes the statistical comparison of quantitative metrics calculated between these four training strategies separately for each center. The same pattern of quantitative metrics in Table 2 is repeated for each center and all metrics across the different frameworks.

**Table 2:** Statistical comparison of quantitative metrics between the four training strategies used in this study. Sequential federated learning (FL-SQ) and parallel federated learning (FL-PL), centralized (CZ), and center based (CB)

	CB		CZ		FL_SQ		FL_PL	
	Mean	95% CI	Mean	95% CI	Mean	95% CI	Mean	95% CI
MAE	0.21 ± 0.07	0.19 to 0.22	0.10 ± 0.03	0.09 to 0.11	0.14 ± 0.07	0.12 to 0.15	0.14 ± 0.06	0.12 to 0.15
ME	− 0.03 ± 0.12	− 0.06 to − 0.003	− 0.01 ± 0.06	− 0.02 to 0.01	− 0.10 ± 0.09	− 0.12 to − 0.07	− 0.10 ± 0.09	− 0.12 to − 0.07
PSNR	28.66 ± 2.70	27.95 to 29.35	34.77 ± 2.56	34.11 to 35.43	33.17 ± 3.53	32.25 to 34.08	33.11 ± 3.49	32.20 to 34.00
ARE (%)	24.22 ± 7.28	22.34 to 26.10	11.16 ± 3.24	10.32 to 12.00	13.51 ± 5.04	12.21 to 14.81	12.83 ± 3.91	11.82 to 13.84
RE (%)	4.70 ± 11.47	1.73 to 7.66	2.05 ± 6.07	0.48 to 3.61	− 6.01 ± 9.18	− 8.38 to 3.63	− 5.64 ± 8.04	− 7.71 to 3.55
SSIM	0.70 ± 0.04	0.68 to 0.70	0.93 ± 0.01	0.92 to 0.93	0.92 ± 0.03	0.91 to 0.93	0.93 ± 0.01	0.92 to 0.93

**Table 3:** Comparison of various image quality metrics (mean ± SD) for the different training models performed at the different centers

		Center 1	Center 2	Center 3	Center 4	Center 5	Center 6
Center based	MAE	0.18 ± 0.03	0.23 ± 0.07	0.19 ± 0.04	0.24 ± 0.07	0.23 ± 0.11	0.19 ± 0.03
	ME	0 ± 0.08	− 0.06 ± 0.12	0 ± 0.09	− 0.09 ± 0.13	− 0.03 ± 0.18	− 0.03 ± 0.09
	PSNR	29.79 ± 1.56	27.87 ± 3.96	29.45 ± 1.94	27.52 ± 3.28	28.16 ± 2.87	29.16 ± 1.53
	ARE (%)	21.96 ± 4.64	24.45 ± 5.92	25 ± 7.07	25.34 ± 9.19	27.42 ± 10.79	21.18 ± 3.19
	RE (%)	7.24 ± 9.71	2.82 ± 11.22	8.63 ± 10.75	0.71 ± 10.83	5.6 ± 16.92	3.21 ± 8.66
	SSIM	0.71 ± 0.03	0.69 ± 0.03	0.71 ± 0.03	0.7 ± 0.06	0.69 ± 0.03	0.69 ± 0.04
Centralized	MAE	0.09 ± 0.02	0.11 ± 0.04	0.1 ± 0.02	0.12 ± 0.04	0.12 ± 0.05	0.09 ± 0.01
	ME	0.01 ± 0.04	− 0.01 ± 0.08	0.01 ± 0.04	− 0.04 ± 0.07	− 0.01 ± 0.09	− 0.02 ± 0.04
	PSNR	35.75 ± 1.59	34.01 ± 3.45	35.53 ± 1.99	33.73 ± 3.4	34.06 ± 2.66	35.55 ± 1.05
	ARE (%)	10.37 ± 2.62	11.8 ± 3.77	11.63 ± 3.29	11.26 ± 2.84	12.48 ± 4.6	9.44 ± 1.11
	RE (%)	3.65 ± 4.9	1.53 ± 7.45	4.38 ± 5.2	− 0.59 ± 5.84	2.55 ± 7.93	0.77 ± 4.34
	SSIM	0.93 ± 0.01	0.93 ± 0.01	0.93 ± 0.01	0.93 ± 0.01	0.93 ± 0.01	0.93 ± 0.01
Federated SQ	MAE	0.14 ± 0.05	0.16 ± 0.11	0.13 ± 0.04	0.15 ± 0.06	0.14 ± 0.06	0.12 ± 0.04
	ME	− 0.08 ± 0.10	− 0.14 ± 0.13	− 0.07 ± 0.10	− 0.12 ± 0.09	− 0.08 ± 0.10	− 0.09 ± 0.06
	PSNR	33 ± 3.15	32.01 ± 5.39	33.98 ± 2.98	32.88 ± 3.4	33.17 ± 2.99	33.97 ± 3.24
	ARE (%)	14.51 ± 7.67	14.1 ± 6.29	13.72 ± 3.3	13.23 ± 4.46	14.29 ± 4.6	11.21 ± 2.8
	RE (%)	− 4.81 ± 12.9	− 9.26 ± 8.67	− 3.36 ± 10.2	− 8.27 ± 7.61	− 3.82 ± 9.7	− 6.53 ± 4.94
	SSIM	0.91 ± 0.07	0.93 ± 0.01	0.93 ± 0.01	0.93 ± 0.01	0.92 ± 0.01	0.93 ± 0.01
Federated PL	MAE	0.13 ± 0.04	0.16 ± 0.11	0.13 ± 0.04	0.15 ± 0.06	0.14 ± 0.06	0.12 ± 0.04
	ME	− 0.09 ± 0.08	− 0.13 ± 0.13	− 0.07 ± 0.09	− 0.12 ± 0.08	− 0.08 ± 0.10	− 0.1 ± 0.05
	PSNR	33.43 ± 2.89	31.82 ± 5.34	33.9 ± 2.97	32.67 ± 3.34	33.1 ± 3.01	33.71 ± 3.26
	ARE (%)	12.22 ± 3.16	13.6 ± 5.69	13.35 ± 2.96	12.85 ± 3.95	13.95 ± 4.49	11.02 ± 2.6
	RE (%)	− 5.54 ± 8.5	− 8.49 ± 8.2	− 2.82 ± 9.59	− 7.65 ± 7.25	− 3.38 ± 9.4	− 5.94 ± 4.89
	SSIM	0.93 ± 0.01	0.93 ± 0.01	0.93 ± 0.01	0.93 ± 0.01	0.92 ± 0.01	0.93 ± 0.01

**Fig. 4:**
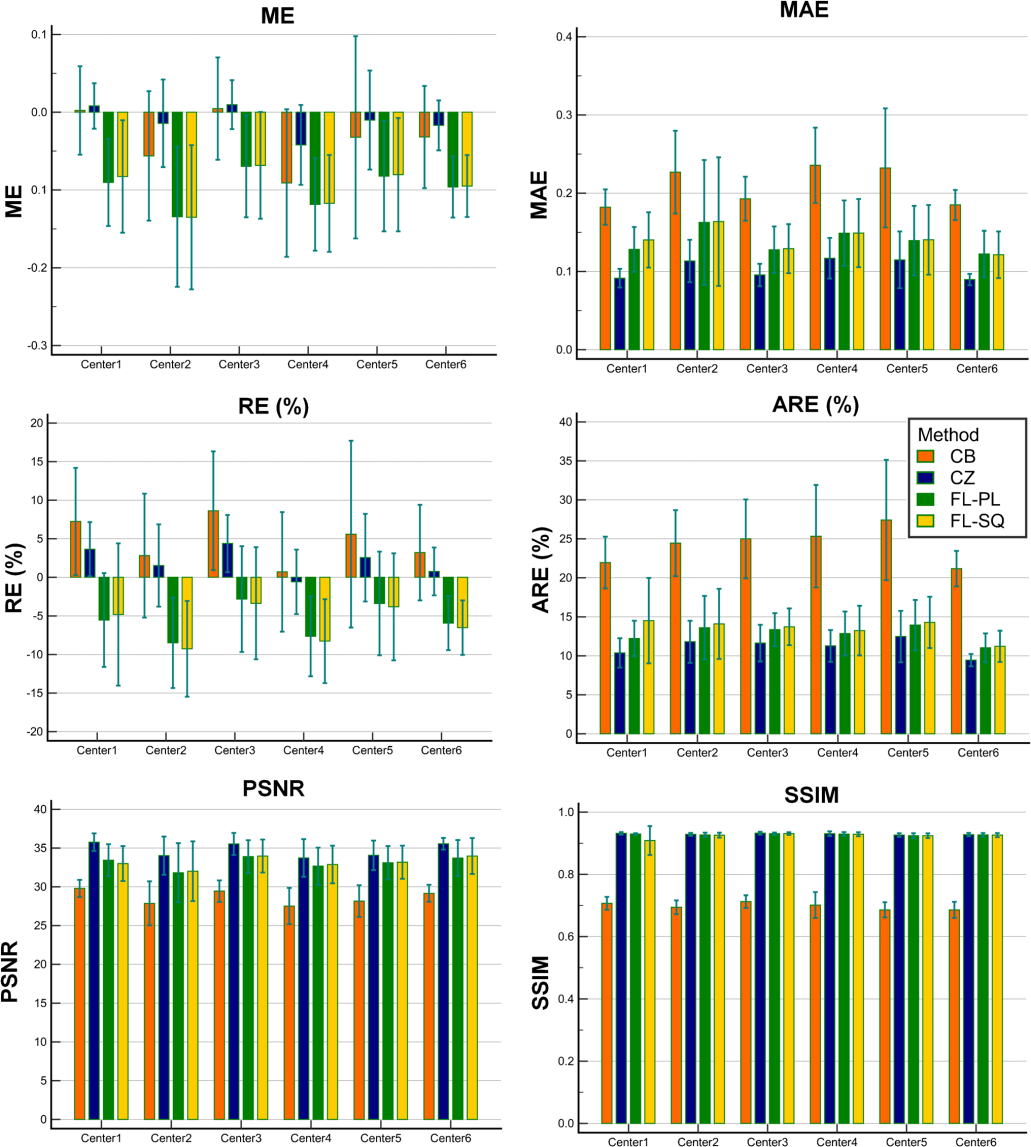
Quantitative performance of the different training strategies, including sequential federated learning (FL-SQ) and parallel federated learning (FL-PL), centralized (CZ), and center based (CB), categorized by clinical center

The quantitative performance of the different training strategies categorized by the clinical center is reported in Fig. 4 (Supplemental Fig. 2 depicts similar information for each patient). The center-wise relative error for the CZ approach (ARE = 11.16 ± 3.24%) demonstrates slightly better performance compared to FL-SQ (ARE = 13.51 ± 5.04%) and FL-PL (ARE = 12.83 ± 3.91%) approaches. Conversely, ARE metric for the CB approach was larger (24.22 ± 7.28%). The highest MAE was achieved by the CB method (0.21 ± 0.07) compared to CZ, FL-SQ, and FL-PL which achieved values of 0.10 ± 0.03, 0.14 ± 0.07, and 0.14 ± 0.06, respectively. For all approaches, SSIM and PSNR metrics demonstrated a consistent behavior over the different centers (0.93 ± 0.02 and 34.0 ± 3.23, respectively), except for CZ which achieved the poorest performance in terms of structural analysis, resulting in SSIM of 0.70 ± 0.04 and PSNR of 28.66 ± 2.70. Supplemental Fig. 2 depicts the quantitative performance of the different training strategies categorized by the different cases in the test dataset.

**Fig. 5:**
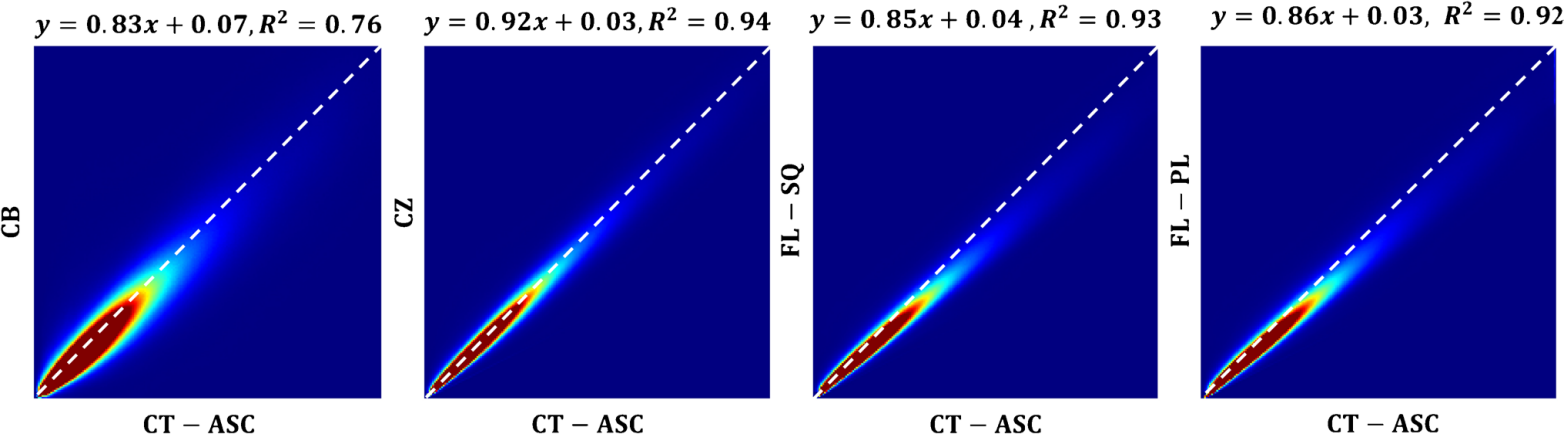
Voxelwise joint histogram analysis depicting the correlation between the predicted images using the different training approaches and CT-ASC images serving as ground truth

Furthermore, the voxelwise joint histogram analysis depicting the correlation between the predicted and CT-ASC images serving as ground truth is illustrated in Fig. 5. The coefficient of determination (*R*^2^) achieved by CB, CZ, FL-Sq, and FL-PL methods were 0.76, 0.94, 0.93, and 0.92, respectively.

**Fig. 6:**
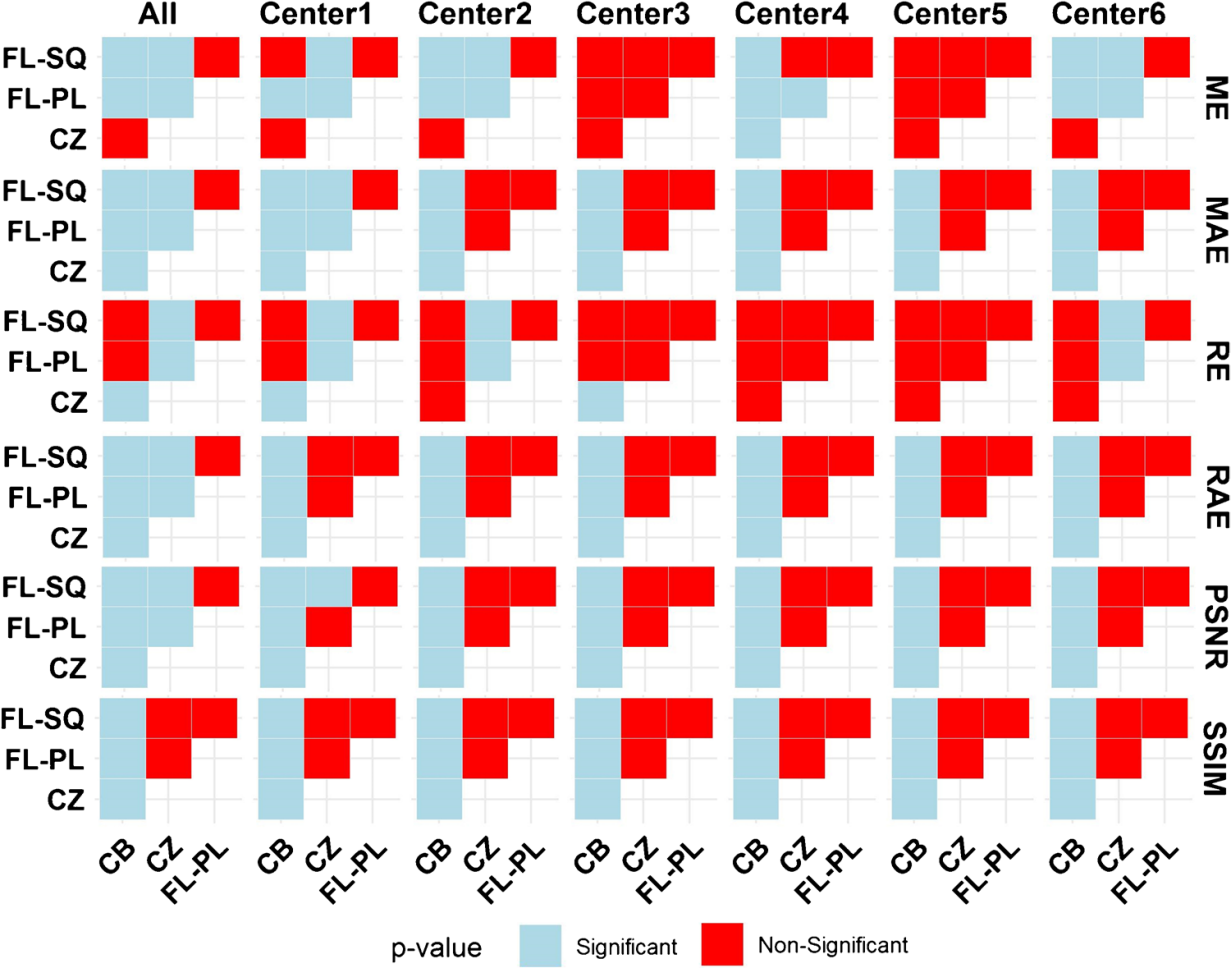
Statistical analysis between the different learning strategies in the form of CB as well as centralized CZ and FL approaches for the different quantitative metrics reflecting evaluation on the overall data as well as on data from each center. Blue and red colors indicate *p*-value < 0.05 and *p*-value > 0.05, respectively. Abbreviations: sequential (FL-SQ) and parallel federated learning (FL-PL), centralized (CZ), and center-based (CB) learning

The results of the statistical analysis between the different learning strategies in the form of center-based categorization are summarized in Fig. 6. As illustrated, the CB approach is significantly different from the other algorithms as reflected by almost all quantitative parameters, except for RE and ME. The CZ model performance demonstrates consistent behavior against FL algorithms in almost all parameters in center-based categorization (*p*-value > 0.05).

## Discussion

DL approaches are data-hungry algorithms that require large, reliable datasets to generate robust and generalizable models [27, 28]. However, the collection of large, centralized datasets for training DL models is challenging and not always feasible owing to the sensitivity of clinical datasets and specifically medical images [27, 28]. FL algorithms provide the opportunity to train a model using multicentric datasets without sharing data [27, 28]. In this work, we provide a framework for DL-based AC/SC model generation from PET images from different centers without the direct sharing of clinical datasets. Our FL-based DL models provided promising results which could improve model generalizability and robustness for AC/SC of PET images without sharing dataset in multicentric studies.

The quantitative analysis performed on SUV PET images demonstrated highly reproducible performance against intra/inter-patient variability. In terms of SUV quantification bias, the ARE% metric demonstrated excellent agreement between FL-SQ (CI:12.21–14.81%) and FL-PL (CI:11.82–13.84%) models and conventional centralized training approach (CI:10.32–12.00%), while FL-based algorithms improved model performance in terms of ARE by more than 11% compared to CB training strategy (CI: 22.34–26.10%). The center-based voxel-wise quantitative analysis and structural indices (Figs. 3 and 4) illustrated the superior performance of FL-based algorithms compared to the CB approach. Furthermore, although in the center-categorized mode, the Mann–Whitney test between different strategies (Fig. 6) revealed consistency between CZ and FL-based algorithms (*p*-value > 0.05) on the overall dataset, the statistical analysis demonstrated significant differences between the different training approaches (*p*-value < 0.05). In addition, the joint histogram analysis (Fig. 5), depicting a voxel-wise comparison between reference CT-ASC and predicted images, exhibited close performance between CZ (*R*^2^ = 0.94), FL-SQ (*R*^2^ = 0.93), and FL-PL (*R*^2^ = 0.92), while the CB model achieved a far lower coefficient of determination (*R*^2^ = 0.74). Despite the strong correlation coefficient between CZ and FL-based methods compared to reference CT-ASC, a slight underestimation of the predicted tracer uptake was observed. Even though slightly inferior results were observed in some CB models, which could be attributed to the different number of slices used in the training of the models (as reflected in Table 1). Overall, all CB models exhibited very similar values of quantitative errors.

In a previous study in the context of conventional centralized learning [20], direct AC/SC of PET images using a modified ResNet algorithm achieved good performance (MAE = 0.22 ± 0.09 and ARE = 11.61 ± 4.25%) using a large dataset (1150 patient images), as gathered from one center and single PET scanner [20]. We then further improved the performance of our algorithm by developing a modified bilevel nested U-NET architecture inspired by U^2^-Net applied for object detection from natural images (centralized mode: MAE = 0.10 ± 0.03 and ARE = 11.16 ± 3.24%). In our previous study [20], DL-based attenuation and scatter correction in the image domain were extensively evaluated on 150 clinical cases including quantitative analysis of radiotracer uptake in 170 lesions/abnormal high-uptake regions (colorectal, head and neck, lung, lymphoma, …). A mean relative SUV error of less than 5% was observed for SUV_max_ and SUV_mean_ across all lesions/regions. Although the quantitative analysis was not performed on malignant lesions in the current study, the voxel-wise SUV error for CZ and FL algorithms was within the same range as our previous study [20].

In comparison to previous works, Yang et al. [63] reported an average ARE of 16.55 ± 4.43% for AC/SC of whole-body PET images using 3D generative adversarial networks. Dong et al. [64] tested different network architectures (U-Net, GAN, and cycle-GAN) achieving good performance in terms of ME (0.62 ± 1.26%) and normalized mean square error (0.72 ± 0.34%), respectively. Van Hemmen et al. [65] developed a modified U-Net architecture for AC/SC of whole body PET images only images resulting in an average ARE of 28.2% on a small-scale dataset. Hwang et al. [66] compared different PET attenuation correction approaches using emission data, including DL-based μ-map generation from non-attenuation-corrected (NAC) images, improved estimation of μ-maps using maximum likelihood estimation of activity and attenuation (MLAA) and a combination of these two methods. They reported that the combination of the MLAA algorithm and DL approach outperformed μ-maps estimated from NAC PET images, whereas no improvement was observed when combining these two approaches. Apart from direct AC/SC on PET images, a number of studies reported on MRI-guided AC/SC by generating pseudo-CT images from PET/MR images and attenuation maps based on tissue classification from PET-only images [67–69]. Although the synthesized attenuation map-based approaches demonstrate promising results, they suffer from numerous challenges, including a mismatch between anatomical (MRI) and PET images and organ motion [67–69]. However, the direct AC/SC approach is less sensitive to noise, metal artifacts, truncation, and local mismatch between anatomical and functional images [70, 71]. Furthermore, this approach is potentially capable of correcting for organ motion and hollow artifacts provided that the model is trained on a clean and accurately corrected PET images [20, 70].

Although direct attenuation/scatter correction in the image domain has a number of advantages, the generation of pseudo µ-maps (synthetic CT) from non-attenuation corrected images or MR images would provide an explainable AC map to verify/detect errors/drawbacks within PET attenuation and scatter correction procedures [20, 66, 72, 73]. The suboptimal performance of direct AC approaches cannot be easily depicted from the resulting PET-AC images (local under/over estimation of radiotracer uptake). However, the suboptimal performance of DL-based synthetic CT generation approaches could be visually detected from the resulting synthetic µ-maps. The resulting synthetic CT images could be visually checked to detect any possible anatomical defects and/or artifacts prior to PET attenuation correction. The other drawback of direct AC in the image domain is the sensitivity of the models to the quality of the query data wherein increased levels of noise, abnormalities, and minor image artifacts may result in erroneous signals in the resulting images. Moreover, the occurrence of outliers (cases with gross errors) in the outcome of these models should be carefully monitored (owing to black-box nature of DL models).

Different studies have been recently performed to assess the performance of FL approaches in medical image analysis [74]. In a study by Feki et al.[36], COVID-19 detection from chest X-ray images using VGG16 and ResNet50 tested federated and centralized frameworks, reporting similar performance for both models. In a more recent study [37], an FL-based model, referred to as EXAM, was developed based on vital signs laboratory exams and chest X-rays for future oxygen requirements of COVID-19 patients across twenty centers. They compared the FL-based model with the center-based model (where each center developed and evaluated the model separately) achieving 16% and 38% improvement in average AUC and generalizability, respectively. Gawali et al. [75] compared different privacy-preserving DL methods for chest X-ray classification tasks. They reported an AUROC of 0.95/0.72 and an F1 score of 0.93/0.62 for a DenseNet model trained in a centralized way and their best-performing FL approach, respectively. In our study, we evaluated two FL-based models and achieved better and comparable results for CB and CZ models, respectively. Building a generalizable and robust model requires a large dataset, while privacy concerns could be addressed by FL approaches without sacrificing models’ performance. In a more recent study, Shiri et al. [76] proposed a FL based multi-institutional PET image segmentation framework on head and neck studies. They enrolled 404 patients from eight different centers and reported that FL-based algorithms outperformed CB and achieved similar performance as the CZ approach.

The FL paradigm enables the training of machine/DL models on multiple decentralized datasets without the need for exchanging data. This preserves data privacy, data security, and data access rights while allowing access to the large-scale heterogeneous database for model training [77]. In the FL framework, the local data are not made available to other participants, or on the server. However, a curious server may infer sensitive data from the exchanged model parameters. In the literature, various attacks have been investigated against machine learning models [78, 79]. For instance, Shokri et al. [80] studied membership inference attacks, whereas Fredrikson et al. [81] addressed model inversion attacks. Possible threats can be classified into three categories, depending on the stage of the process of an FL system. Malicious parties can perform data poisoning attacks at the “input” of the learning model [82, 83]. For instance, they may modify the label of the data samples. Alternatively, they can perform model poisoning attacks during the learning process [84, 85]. For example, they may upload random updates to the global model. Finally, a malicious party can perform inference attacks on the “released learnt model” [77, 80, 81]. For instance, a curious server may infer sensitive information about the training data from the communicated model parameters.

CB framework faces generalizability challenges even with large datasets owing to the large variability across different centers in terms of scanner brands, data acquisition and reconstruction protocols, and post-processing schemes. Moreover, due to the absence of infrastructures and expertise, it may not possible to build ML models at each center. The CZ training framework is the ideal option for ML model development. Yet, it suffers from limitations imposed by ethical and legal constraints. FL algorithms provide the opportunity to train a model using multicentric datasets without sharing data, and models trained with the FL framework can converge to the CZ performance in the ideal situation. Overall, the CZ model training would lead to the highest accuracy and generalizability. However, in cases where ethical and legal constraints do not allow data sharing or when a center does not have enough training samples FL approaches would be an attractive solution. Models trained with FL in the best-case scenario might approach the performance of the CZ models. On the other hand, CB models are observed to suffer from very poor generalizability.

Data heterogeneity arising from the use of different scanners, image acquisition and reconstruction protocols, is the main source of error impairing building a generalizable model [17]. The heterogeneity of data across the different centers prevents CB models from working properly on an unseen dataset from the other centers. To build a generalizable model, data from different centers should be included in the training dataset, which is possible in CZ and FL pipelines. In FL, a global model is built based on a portion of the data from the different centers in a federated approach and then for each center, the global model will be specialized for each center by applying a transfer learning technique using a transfer-FL framework [27, 28]. This approach could be employed to comply with the heterogeneous data collected from the different centers with various acquisition parameters.

This study inherently bears a number of limitations. The implementation of all models was performed on a server using different GPUs where the different nodes were considered as centers similar to previous FL studies [35, 36, 54–59, 74, 76]. The challenges of FL, such as local computer capacity, and communication bottleneck between centers and local server should be considered in the real clinical scenario. Further studies should be performed in real clinical situations using a larger size of the training dataset. In the current version, a proof-of-concept has been demonstrated and further investigation with larger cohorts is warranted. One limitation of FL is data preparation and preprocessing due to the nature of the decentralized process. However, for image preprocessing, including normalization, we used an easy method to ensure reproducibility.

## Conclusion

AC/SC are key corrections required to enable quantitative PET imaging, which remains challenging on CT-less PET scanners (PET/MRI and standalone PET-only scanners). DL-based models provide very promising results and might outperform conventional algorithms in terms of attenuation and scatter corrections. At the same time, robust and generalizable DL models require heterogeneous, large, and reliable datasets from multiple centers. Yet, legal/ethical/privacy considerations prevent the collection of very large datasets. In this work, we developed an FL-based framework for anatomical knowledge free or CT-less AC/SC of PET images, which proved to outperform center-based models, demonstrating comparable performance with respect to centralized DL. FL-based DL provided promising results through improving model generalizability and robustness for AC/SC of PET images without direct sharing of datasets amongst centers.

## Supplementary Information

Below is the link to the electronic supplementary material.

Supplementary file1 (PDF 513 kb)

## Data Availability

Data are available from The Cancer Imaging Archive (TCIA) from refereces of [20, 39–47].

## References

[1] Blodgett TM, Meltzer CC, Townsend DW (2007). PET/CT: Form and function. Radiology.

[2] Zaidi H, Montandon ML, Meikle S (2007). Strategies for attenuation compensation in neurological PET studies. Neuroimage.

[3] Zaidi H, Karakatsanis N (2018). Towards enhanced PET quantification in clinical oncology. Br J Radiol.

[4] Zaidi H, Hasegawa B (2003). Determination of the attenuation map in emission tomography. J Nucl Med.

[5] Zaidi H, Koral KF (2004). Scatter modelling and compensation in emission tomography. Eur J Nucl Med Mol Imaging.

[6] Zaidi H, Montandon ML (2007). Scatter compensation techniques in PET. PET Clin.

[7] Akbarzadeh A, Ay MR, Ahmadian A, Alam NR, Zaidi H (2013). MRI-guided attenuation correction in whole-body PET/MR: assessment of the effect of bone attenuation. Ann Nucl Med.

[8] Mehranian A, Arabi H, Zaidi H (2016). Vision 20/20: Magnetic resonance imaging-guided attenuation correction in PET/MRI: challenges, solutions, and opportunities. Med Phys.

[9] Berker Y, Li Y (2016). Attenuation correction in emission tomography using the emission data–a review. Med Phys.

[10] Arabi H, Zaidi H (2020). Deep learning-guided estimation of attenuation correction factors from time-of-flight PET emission data. Med Image Anal.

[11] Shiri I, Arabi H, Sanaat A, Jenabi E, Becker M, Zaidi H (2021). Fully automated gross tumour volume delineation from PET in head and neck cancer using deep learning algorithms. Clin Nucl Med.

[12] Yousefirizi F, Decasez P, Amyar A, Ruan S, Saboury B, Rahmim A. Artificial intelligence-based detection, classification and prediction/prognosis in PET imaging: towards radiophenomics. arXiv preprint arXiv:211010332. 2021.

[13] Mohammadi R, Shokatian I, Salehi M, Arabi H, Shiri I, Zaidi H (2021). Deep learning-based auto-segmentation of organs at risk in high-dose rate brachytherapy of cervical cancer. Radiother Oncol.

[14] Salimi Y, Shiri I, Akhavanallaf A, Mansouri Z, Saberi Manesh A, Sanaat A (2021). Deep learning-based fully automated Z-axis coverage range definition from scout scans to eliminate overscanning in chest CT imaging. Insights Imaging.

[15] Sanaat A, Shooli H, Ferdowsi S, Shiri I, Arabi H, Zaidi H (2021). DeepTOFSino: a deep learning model for synthesizing full-dose time-of-flight bin sinograms from their corresponding low-dose sinograms. Neuroimage.

[16] Sanaat A, Akhavanalaf A, Shiri I, Salimi Y, Arabi H, Zaidi H (2022). Deep-TOF-PET: Deep learning-guided generation of time-of-flight from non-TOF brain PET images in the image and projection domains. Hum Brain Mapp.

[17] Jabbarpour A, Mahdavi SR, Vafaei Sadr A, Esmaili G, Shiri I, Zaidi H (2022). Unsupervised pseudo CT generation using heterogenous multicentric CT/MR images and CycleGAN: dosimetric assessment for 3D conformal radiotherapy. Comput Biol Med.

[18] Armanious K, Hepp T, Küstner T, Dittmann H, Nikolaou K, La Fougère C (2020). Independent attenuation correction of whole body [(18)F]FDG-PET using a deep learning approach with Generative Adversarial Networks. EJNMMI Res.

[19] Qian H, Rui X, Ahn S. Deep learning models for PET scatter estimations. 2017 IEEE Nuclear Science Symposium and Medical Imaging Conference (NSS/MIC); 2017. p. 1–5.

[20] Shiri I, Arabi H, Geramifar P, Hajianfar G, Ghafarian P, Rahmim A (2020). Deep-JASC: joint attenuation and scatter correction in whole-body (18)F-FDG PET using a deep residual network. Eur J Nucl Med Mol Imaging.

[21] McMillan AB, Bradshaw TJ (2021). Artificial Intelligence-based data corrections for attenuation and scatter in position emission tomography and single-photon emission computed tomography. PET Clin.

[22] Rieke N, Hancox J, Li W, Milletarì F, Roth HR, Albarqouni S (2020). The future of digital health with federated learning. NPJ Digit Med.

[23] Kaissis GA, Makowski MR, Rückert D, Braren RF (2020). Secure, privacy-preserving and federated machine learning in medical imaging. Nat Mach Intell.

[24] Kirienko M, Sollini M, Ninatti G, Loiacono D, Giacomello E, Gozzi N (2021). Distributed learning: a reliable privacy-preserving strategy to change multicenter collaborations using AI. Eur J Nucl Med Mol Imaging.

[25] Navid Hasani MAM, Arman Rhamim, Ronald M. Summers, Elizabeth Jones, Eliot Siegel, Babak Saboury. Trustworthy artificial intelligence in medical imaging. PET Clin. 2021:17:1–12. 10.1016/j.cpet.2021.09.007 PMC8785402 34809860

[26] Li Q, Wen Z, Wu Z, Hu S, Wang N, Li Y, et al. A survey on federated learning systems: vision, hype and reality for data privacy and protection. arXiv preprint arXiv:190709693. 2019.

[27] Jorge VAM, Granada R, Maidana RG, Jurak DA, Heck G, Negreiros APF, et al. A survey on unmanned surface vehicles for disaster robotics: main challenges and directions. Sensors (Basel). 2019;19. 10.3390/s19030702. 10.3390/s19030702 PMC6387351 30744069

[28] Shyu C-R, Putra KT, Chen H-C, Tsai Y-Y, Hossain KT, Jiang W (2021). A systematic review of federated learning in the healthcare area: from the perspective of data properties and applications. Appl Sci.

[29] Konečný J, McMahan HB, Yu FX, Richtárik P, Suresh AT, Bacon D. Federated learning: strategies for improving communication efficiency. arXiv preprint arXiv:161005492. 2016.

[30] Singh A, Vepakomma P, Gupta O, Raskar R. Detailed comparison of communication efficiency of split learning and federated learning. arXiv preprint arXiv:190909145. 2019.

[31] Luping W, Wei W, Bo L. CMFL: Mitigating communication overhead for federated learning. 2019 IEEE 39th International Conference on Distributed Computing Systems (ICDCS): 2019. p. 954–64.

[32] Amiri MM, Gunduz D, Kulkarni SR, Poor HV. Federated learning with quantized global model updates. arXiv preprint arXiv:200610672. 2020.

[33] Li W, Milletarì F, Xu D, Rieke N, Hancox J, Zhu W, et al. Privacy-preserving federated brain tumour segmentation. International workshop on machine learning in medical imaging: Springer; 2019. p. 133–41. 10.1007/978-3-030-32692-0_16 PMC7616593 39453415

[34] Xia Y, Yang D, Li W, Myronenko A, Xu D, Obinata H, et al. Auto-FedAvg: learnable federated averaging for multi-institutional medical image segmentation. arXiv preprint:210410195. 2021.

[35] Shiri I, Amini M, Salimi Y, Sanaat A, Saberi A, Razeghi B, et al. Multi-institutional PET/CT image segmentation using a decentralized federated deep transformer learning algorithm. J Nucl Med; 2022;63(Suppl2):3348.

[36] Feki I, Ammar S, Kessentini Y, Muhammad K (2021). Federated learning for COVID-19 screening from Chest X-ray images. Appl Soft Comput.

[37] Dayan I, Roth HR, Zhong A, Harouni A, Gentili A, Abidin AZ (2021). Federated learning for predicting clinical outcomes in patients with COVID-19. Nat Med.

[38] Roth HR, Chang K, Singh P, Neumark N, Li W, Gupta V, et al. Federated learning for breast density classification: a real-world implementation. In: Domain Adaptation and Representation Transfer, and Distributed and Collaborative Learning: Lecture Notes in Computer Science, Vol. 12444. Springer, Cham. 2020. pp. 181–91. 10.1007/978-3-030-60548-3_18

[39] Clark K, Vendt B, Smith K, Freymann J, Kirby J, Koppel P (2013). The Cancer Imaging Archive (TCIA): maintaining and operating a public information repository. J Digit Imaging.

[40] Machtay M, Duan F, Siegel BA, Snyder BS, Gorelick JJ, Reddin JS (2013). Prediction of survival by [18F]fluorodeoxyglucose positron emission tomography in patients with locally advanced non-small-cell lung cancer undergoing definitive chemoradiation therapy: results of the ACRIN 6668/RTOG 0235 trial. J Clin Oncol.

[41] Kinahan P, Muzi M, Bialecki B, Herman B, Coombs L. Data from the ACRIN 6668 Trial NSCLC-FDG-PET. Cancer Imaging Arch. 2019;10. 10.7937/tcia.2019.30ilqfcl

[42] Bakr S, Gevaert O, Echegaray S, Ayers K, Zhou M, Shafiq M (2017). Data for NSCLC radiogenomics collection. The Cancer Imaging Archive.

[43] Bakr S, Gevaert O, Echegaray S, Ayers K, Zhou M, Shafiq M (2018). A radiogenomic dataset of non-small cell lung cancer. Sci Data.

[44] Gevaert O, Xu J, Hoang CD, Leung AN, Xu Y, Quon A (2012). Non-small cell lung cancer: identifying prognostic imaging biomarkers by leveraging public gene expression microarray data–methods and preliminary results. Radiology.

[45] Grossberg A, Elhalawani H, Mohamed A, Mulder S, Williams B, White A, et al. MD Anderson Cancer Center Head and Neck Quantitative Imaging Working Group.(2020) HNSCC . The Cancer Imaging Archive. doi:107937/k9/tcia. 2020:a8sh-7363.

[46] Grossberg AJ, Mohamed ASR, Elhalawani H, Bennett WC, Smith KE, Nolan TS (2018). Imaging and clinical data archive for head and neck squamous cell carcinoma patients treated with radiotherapy. Sci Data.

[47] Matched computed tomography segmentation and demographic data for oropharyngeal cancer radiomics challenges. Sci Data. 2017;4:170077. 10.1038/sdata.2017.77. 10.1038/sdata.2017.77 PMC5497772 28675381

[48] Bonawitz K, Eichner H, Grieskamp W, Huba D, Ingerman A, Ivanov V, et al. Towards federated learning at scale: system design. arXiv preprint arXiv:190201046. 2019.

[49] Li T, Sahu AK, Talwalkar A, Smith V (2020). Federated learning: Challenges, methods, and future directions. IEEE Signal Process Mag.

[50] Amiri MM, Gündüz D (2020). Federated learning over wireless fading channels. IEEE Trans Wirel Commun.

[51] Wei K, Li J, Ding M, Ma C, Yang HH, Farokhi F (2020). Federated learning with differential privacy: algorithms and performance analysis. IEEE Trans Inf Forensics Secur.

[52] Mothukuri V, Parizi RM, Pouriyeh S, Huang Y, Dehghantanha A, Srivastava G (2021). A survey on security and privacy of federated learning. Future Gener Comput Syst.

[53] Lu Y, Huang X, Dai Y, Maharjan S, Zhang Y (2019). Blockchain and federated learning for privacy-preserved data sharing in industrial IoT. IEEE Trans Industr Inform.

[54] Zhang M, Qu L, Singh P, Kalpathy-Cramer J, Rubin DL. SplitAVG: A heterogeneity-aware federated deep learning method for medical imaging. arXiv preprint arXiv:210702375. 2021. 10.1109/JBHI.2022.3185956 PMC9749741 35749336

[55] Stripelis D, Saleem H, Ghai T, Dhinagar N, Gupta U, Anastasiou C, et al. Secure neuroimaging analysis using federated learning with homomorphic encryption. 17th International Symposium on Medical Information Processing and Analysis: SPIE; 2021. p. 351–359.

[56] Qu L, Zhou Y, Liang PP, Xia Y, Wang F, Fei-Fei L, et al. Rethinking architecture design for tackling data heterogeneity in federated learning. arXiv preprint arXiv:210606047. 2021. 10.1109/cvpr52688.2022.00982 PMC9826695 36624800

[57] Liu Q, Yang H, Dou Q, Heng P-A. Federated semi-supervised medical image classification via inter-client relation matching. arXiv preprint arXiv:210608600. 2021.

[58] Chakravarty A, Kar A, Sethuraman R, Sheet D. Federated learning for site aware chest radiograph screening. 2021 IEEE 18th International Symposium on Biomedical Imaging (ISBI): IEEE; 2021. p. 1077–81.

[59] Linardos A, Kushibar K, Walsh S, Gkontra P, Lekadir K. Federated learning for multi-center imaging diagnostics: a study in cardiovascular disease. arXiv preprint arXiv:210703901. 2021. 10.1038/s41598-022-07186-4 PMC8894335 35241683

[60] Qin X, Zhang Z, Huang C, Dehghan M, Zaiane OR, Jagersand M (2020). U2-Net: going deeper with nested U-structure for salient object detection. Pattern Recognit.

[61] Ronneberger O, Fischer P, Brox T. U-net: convolutional networks for biomedical image segmentation. International Conference on Medical image computing and computer-assisted intervention: Springer; 2015. p. 234–41.

[62] Wang Z, Bovik AC, Sheikh HR, Simoncelli EP (2004). Image quality assessment: from error visibility to structural similarity. IEEE Trans Image Process.

[63] Yang X, Lei Y, Dong X, Wang T, Higgins K, Liu T, et al. Attenuation and scatter correction for whole-body PET using 3D generative adversarial networks. J Nucl Med; 2019;60(Suppl 1):174.

[64] Dong X, Lei Y, Wang T, Higgins K, Liu T, Curran WJ (2020). Deep learning-based attenuation correction in the absence of structural information for whole-body positron emission tomography imaging. Phys Med Biol.

[65] Van Hemmen H, Massa H, Hurley S, Cho S, Bradshaw T, McMillan A. A deep learning-based approach for direct whole-body PET attenuation correction. J Nucl Med.;60,559, 2019.

[66] Hwang D, Kang SK, Kim KY, Choi H, Lee JS (2022). Comparison of deep learning-based emission-only attenuation correction methods for positron emission tomography. Eur J Nucl Med Mol Imaging.

[67] Liu F, Jang H, Kijowski R, Bradshaw T, McMillan AB (2018). Deep learning MR imaging-based attenuation correction for PET/MR imaging. Radiology.

[68] Liu F, Jang H, Kijowski R, Zhao G, Bradshaw T, McMillan AB (2018). A deep learning approach for (18)F-FDG PET attenuation correction. EJNMMI Phys.

[69] Yang J, Sohn JH, Behr SC, Gullberg GT, Seo Y (2020). CT-less direct correction of attenuation and scatter in the image space using deep learning for whole-body FDG PET: potential benefits and pitfalls. Radiol Artif Intell.

[70] Shiri I, Sanaat A, Salimi Y, Akhavanallaf A, Arabi H, Rahmim A, et al. PET-QA-NET: Towards routine PET image artifact detection and correction using deep convolutional neural networks. 2021 IEEE Nuclear Science Symposium and Medical Imaging Conference (NSS/MIC); p. 1–3. 10.1109/NSS/MIC44867.2021.9875610

[71] Izadi S, Shiri I, Uribe C, Geramifar P, Zaidi H, Rahmim A, et al. Enhanced direct joint attenuation and scatter correction of whole-body PET images via context-aware deep networks. medRxiv. 2022. 10.1101/2022.05.26.2227566210.1016/j.zemedi.2024.01.00238302292

[72] Chen X, Zhou B, Xie H, Shi L, Liu H, Holler W (2022). Direct and indirect strategies of deep-learning-based attenuation correction for general purpose and dedicated cardiac SPECT. Eur J Nucl Med Mol Imaging.

[73] Toyonaga T, Shao D, Shi L, Zhang J, Revilla EM, Menard D (2022). Deep learning-based attenuation correction for whole-body PET - a multi-tracer study with (18)F-FDG, (68) Ga-DOTATATE, and (18)F-Fluciclovine. Eur J Nucl Med Mol Imaging.

[74] Shiri I, Sadr AV, Sanaat A, Ferdowsi S, Arabi H, Zaidi H. Federated learning-based deep learning model for PET attenuation and scatter correction: a multi-center study. 2021 IEEE Nuclear Science Symposium and Medical Imaging Conference (NSS/MIC). p. 1–3.

[75] Gawali M, Arvind C, Suryavanshi S, Madaan H, Gaikwad A, Prakash KB, et al. Comparison of privacy-preserving distributed deep learning methods in healthcare. Annual Conference on Medical Image Understanding and Analysis: Springer; 2021. p. 457–71.

[76] Shiri I, Vafaei Sadr A, Amini M, Salimi Y, Sanaat A, Akhavanallaf A (2022). Decentralized distributed multi-institutional pet image segmentation using a federated deep learning framework. Clin Nucl Med.

[77] Melis L, Song C, De Cristofaro E, Shmatikov V. Exploiting unintended feature leakage in collaborative learning. 2019 IEEE Symposium on Security and Privacy (SP): IEEE; 2019. p. 691–706.

[78] Carlini N, Liu C, Erlingsson Ú, Kos J, Song D. The secret sharer: evaluating and testing unintended memorization in neural networks. 28th Security Symposium ( Security 19); 2019. p. 267–84.

[79] Duchi JC, Jordan MI, Wainwright MJ (2014). Privacy aware learning. Journal of the ACM (JACM).

[80] Shokri R, Stronati M, Song C, Shmatikov V. Membership inference attacks against machine learning models. 2017 IEEE Symposium on Security and Privacy (SP): IEEE; 2017. p. 3–18.

[81] Fredrikson M, Jha S, Ristenpart T. Model inversion attacks that exploit confidence information and basic countermeasures. Proc of the 22nd ACM SIGSAC Conference on Computer and Communications Security; 2015. p. 1322–33.

[82] Chen X, Liu C, Li B, Lu K, Song D. Targeted backdoor attacks on deep learning systems using data poisoning. arXiv preprint arXiv:171205526. 2017.

[83] Li B, Wang Y, Singh A, Vorobeychik Y (2016). Data poisoning attacks on factorization-based collaborative filtering. Adv Neural Inf Process Syst.

[84] Bagdasaryan E, Veit A, Hua Y, Estrin D, Shmatikov V. How to backdoor federated learning. International Conference on Artificial Intelligence and Statistics: PMLR; 2020. p. 2938–48.

[85] Xie C, Huang K, Chen P-Y, Li B. DBA: Distributed backdoor attacks against federated learning. International Conference on Learning Representations; 2020; p1–15.

