# Supplementary material for: Decentralized collaborative multi-institutional PET attenuation and scatter correction using federated deep learning
Source: Eur J Nucl Med Mol Imaging. 2022 Dec 12;50(4):1034–50. doi: 10.1007/s00259-022-06053-8 (PMC9742659; doi:10.1007/s00259-022-06053-8)
Supplement: Supplementary file 1 — Supplementary file1 (PDF 513 kb) [file 259_2022_6053_MOESM1_ESM.pdf]

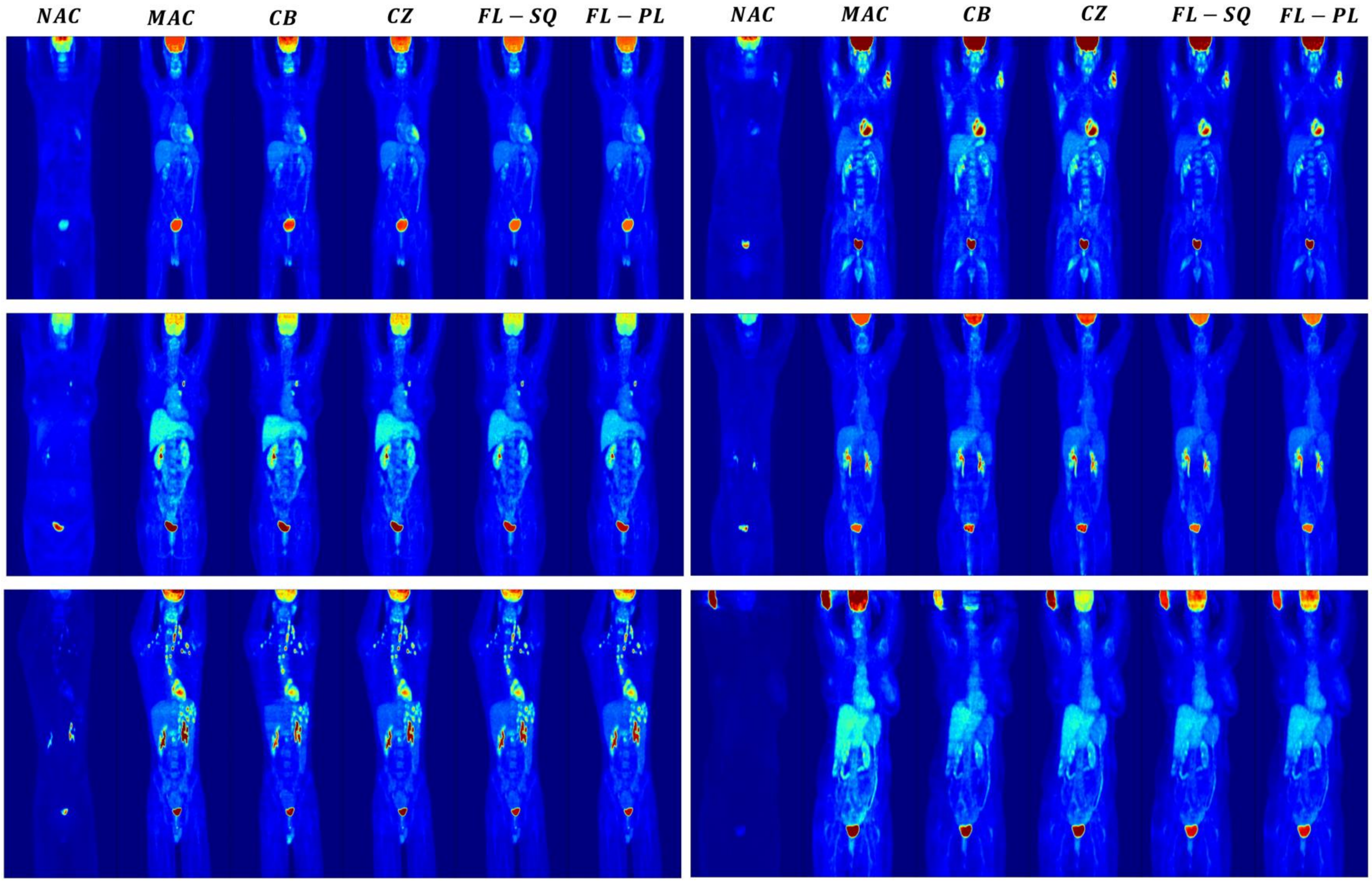

Supplemental Figure 1. Example of non-ASC, CT-ASC, CB base model, CZ base model, FL-SQ based model, and FL-PL model maximum intensity projections for different cases from different clinical centers.

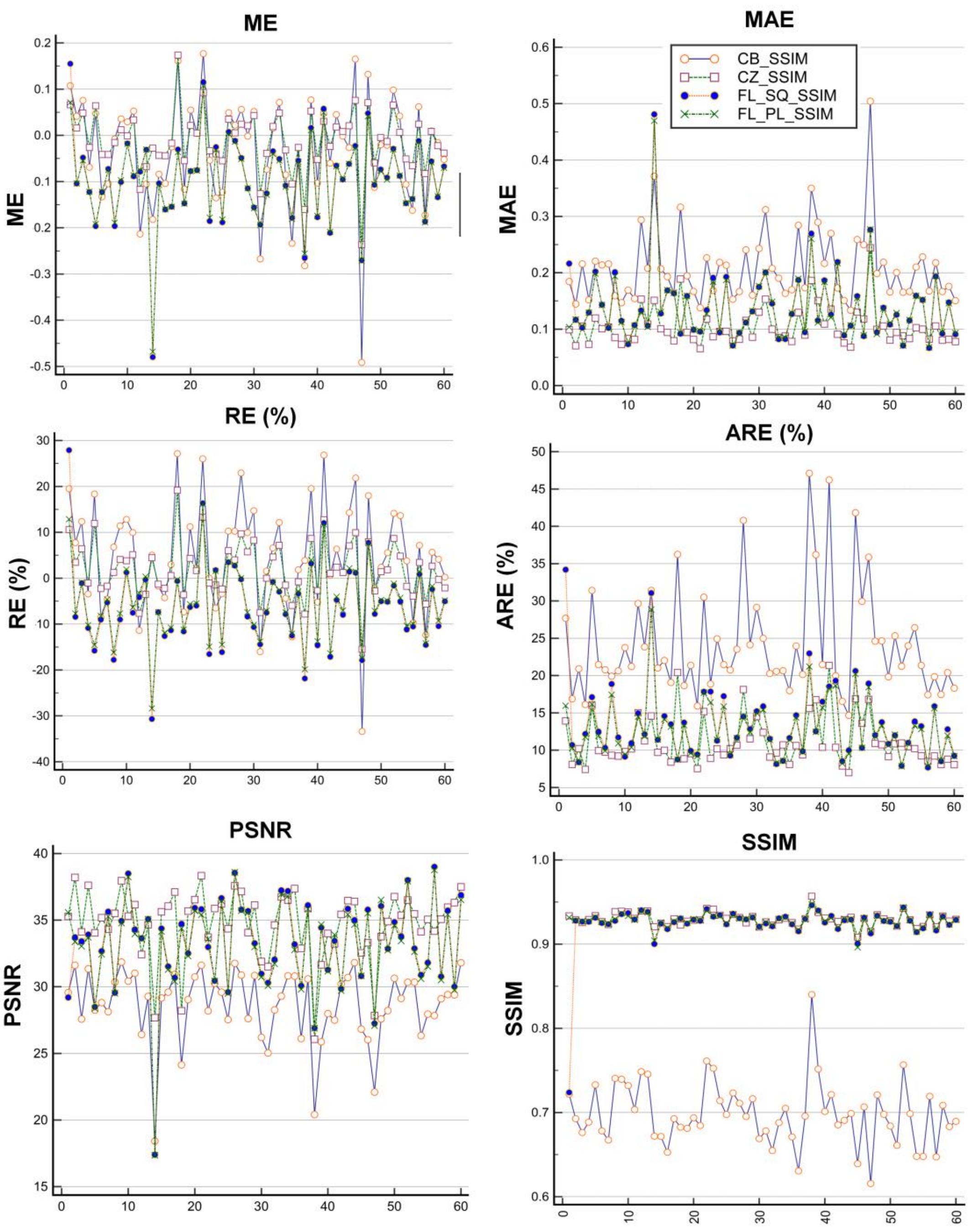

Supplemental Figure 2. Quantitative performance of the different training strategies categorized by case.
